# 成人T淋巴母细胞淋巴瘤诊断与治疗中国专家共识（2023年版）

**DOI:** 10.3760/cma.j.issn.0253-2727.2023.05.001

**Published:** 2023-05

**Authors:** 中国抗癌协会血液肿瘤专业委员会 中华医学会血液学分会 中国抗癌协会血液肿瘤专业委员会T细胞淋巴瘤工作组

T淋巴母细胞淋巴瘤（T-LBL）是一种起源于非成熟前体T淋巴细胞的高度侵袭性肿瘤，恶性程度高，异质性强。年发病率为1～5/10万，占成人非霍奇金淋巴瘤（NHL）的3％～4％，占儿童NHL的40％左右，亚洲的发病率较欧美高，尤以我国和其他东亚国家为著。目前认为T-LBL与急性T淋巴细胞白血病（T-ALL）是具有不同临床表现、处于不同发展阶段的同一类疾病，2022年版《WHO造血与淋巴组织肿瘤分类》将骨髓中原始和幼稚淋巴细胞比例≥25％定义为ALL[1]。T-LBL的治疗目前尚不统一，总体缓解率（ORR）可达70％～90％，3～5年无病生存（DFS）率仅30％～60％，复发后只有不到20％的患者可以长期生存，诊治策略有待进一步规范和完善，因此在中国抗癌协会血液肿瘤专业委员会指导下制定本共识。

一、临床表现

T-LBL的临床表现与T-ALL不尽相同（表1），常伴有前纵隔巨大包块，典型临床表现为前纵隔巨大肿块所致的上腔静脉综合征、气道压迫所致的咳嗽、呼吸困难等，部分患者可伴有胸腔或心包积液，15％～20％的患者初诊时合并骨髓或中枢神经系统（CNS）浸润。部分患者确诊时可表现为白细胞计数增高，大部分患者确诊时合并B症状[2]。

**表1:** 成人T淋巴母细胞淋巴瘤（T-LBL）与急性T淋巴细胞白血病（T-ALL）常见临床特征

临床特征	T-LBL[3]–[4]	T-ALL[5]
中位年龄（岁）	25～33	30
男性（%）	62.5～82.0	70
纵隔包块（%）	70～94	66
浆膜腔积液（%）	30.0～53.4	1
中枢神经系统侵犯（%）	4.6～10.0	7
骨髓侵犯（%）	15～33	100

二、诊断

1. 病理诊断：组织病理是诊断T-LBL的金标准。①细胞形态：T-LBL主要表现为中等大小的肿瘤细胞呈弥漫性生长；细胞核圆形、不规则或扭曲，染色质细，核仁不明显，核分裂象易见；细胞质较少。②免疫组化：主要标志包括CD3、CD1a、CD2、CD4、CD5、CD7、CD8、CD10、CD99、TdT等；90％的病例TdT、CD3、CD7阳性；近半数的病例CD4和CD8同时阳性，CD10、CD34、CD1a及CD99也常呈阳性；同时检测CD33、CD117、MPO、Lysozyme等有助于与急性髓系白血病鉴别，需注意少数T-LBL亦可表达部分髓系标志；纵隔肿块患者，建议增加AE1/AE3和CK19等检测与胸腺瘤鉴别[6]。T-LBL通常LMO2阳性，也有助于和胸腺瘤鉴别。

CD7、CD43不能单独作为T淋巴细胞标志；T-LBL与T-ALL免疫表型基本相同，但T-LBL更倾向于成熟胸腺表型，更少表达髓系抗原[7]。

2. 骨髓细胞学和免疫分型：部分患者合并骨髓侵犯。①细胞形态学：骨髓有核细胞增生活跃或明显活跃，伴原始细胞增生，但比例<25％。瘤细胞形态异常，呈圆形、椭圆形或有尾状突起；细胞核多为圆形，核大，核染色质粗细不均、排列不规则，核可见凹陷、折叠、切迹及裂痕等；细胞质量少，核质比高。②免疫分型：多参数流式细胞术检测，T-LBL表达T细胞特异性抗原CD3（sCD3或cCD3）；常表达CD38、CD7、CD99、cTdT、CD2，不同程度表达CD1a、CD2、CD4、CD5、CD8；共表达CD4、CD8。根据抗原的表达，T-LBL又可分类为：pro-T、pre-T、皮质-T、髓质-T，早期T前体淋巴母细胞白血病（ETP-ALL/LBL）由于具有特殊的细胞形态及免疫表型，应作为建议的分类亚型。

20％～30％的T-ALL/LBL可见髓系标志CD13、CD33。CD117偶尔阳性，可能与FLT3突变相关。不能因此排除T-ALL/LBL诊断，也不能确诊为混合细胞白血病。

3. 细胞遗传学：T-LBL常见主要基因突变：NOTCH1（>50％）[8]、FBXW7（11％～31％）、LOH6q（13％）、PHF6（青少年16％，成人39％）[9]、IL7R、JAK1、JAK3（青少年35％，成人26％）[10]、NRAS/KRAS（成人10％）[11]和PTEN[11]–[12]等。

T-LBL染色体重组特征：9q34重组占10％，主要涉及t（9;17）（q34;q22-23），导致SET、ABL1、NUP214及NOTCH1基因表达异常[13]；19％的T-LBL发生6号染色体杂合缺失（LOH6q），导致GRIK2、CASP8AP2、EPHA7基因表达异常[14]。

T-LBL的细胞遗传学特征：与血管生成、细胞黏附及趋化、肿瘤转移等相关的基因在T-LBL中高表达[15]。

T-LBL与T-ALL的细胞遗传学具有很多相似之处；儿童T-LBL的染色体易位和T-ALL较相似。

4. 分期与预后：所有确诊患者在治疗前均应进行常规生化检查、骨髓穿刺及活检、增强CT或PET/CT以明确患者分期及危险度分层（表2）。成人危险度分层常参考国际预后指数（IPI）（表3），IPI评分0～1分定义为低危组，2～3分定义为中危组，4～5分定义为高危组。

**表2:** Ann Arbor分期

分期	定义
Ⅰ期	单个淋巴结区受累（Ⅰ），或单个结外器官或部位受累且无淋巴结受累（ⅠE）。单个淋巴结区可包括一个淋巴结或一组相邻的淋巴结
Ⅱ期	仅累及横膈同侧的2个或以上淋巴结区（Ⅱ），或以上受累伴局限性相邻单个结外器官或组织受累（ⅡE）
Ⅲ期	横膈两侧的淋巴结区或淋巴结构受累，可伴脾累及（ⅢS），结外器官局限受累（ⅢE），或脾与局限性结外器官受累（ⅢSE）
Ⅳ期	一个或多个结外器官受到广泛性或播散性侵犯，伴或不伴相关淋巴结受累。肝或骨髓受累均为Ⅳ期

**表3:** 国际预后指数（IPI）

IPI评分项目	0分	1分
年龄（岁）	≤60	>60
ECOG评分	0或1级	2～4级
临床分期	Ⅰ或Ⅱ	Ⅲ或Ⅳ
结外受侵部位数目	<2	≥2
LDH	正常	升高

**注** ECOG：美国东部肿瘤协作组；LDH：乳酸脱氢酶

T-LBL预后不良相关临床特征包括Ⅲ/Ⅳ期、美国东部肿瘤协作组体能状况（ECOG-PS）评分≥2、LDH升高、CNS受累等。近年来，针对基因突变、基因表达谱[16]、miRNA表达谱等探索的T-LBL新型分子预后标志受到关注，如有研究发现NOTCH1/FBXW7突变（N/F突变）、PHF6突变等与T-LBL预后良好相关，BRD2表达上调[17]和miR-374b表达降低[18]与T-LBL耐药相关。

三、治疗

1. 治疗原则：所有患者一经确诊均应按照全身性疾病治疗，T-LBL的治疗过程包括诱导治疗、巩固强化、维持治疗等阶段。应根据患者的危险度选择合适的治疗策略和方案。对于年轻的成人患者，参照儿童ALL方案治疗的疗效优于NHL经典方案；对于肝、脾、淋巴结肿大明显，或有发生肿瘤溶解倾向的患者可给予预治疗。预治疗可采用糖皮质激素联合环磷酰胺（CTX）。所有患者均需进行CNS预防性治疗，且应尽早开始。

2. 成人T-LBL治疗方案：由于缺乏特异性的治疗靶点，目前T-LBL的诱导治疗仍以多药联合的治疗方案为主，主要包括的药物如：糖皮质激素（如泼尼松、地塞米松等）、蒽环/蒽醌类药物［如柔红霉素（DNR）、去甲氧柔红霉素（IDA）、阿霉素、米托蒽醌等］、长春新碱（VCR）或长春地辛、门冬酰胺酶（ASP，大肠杆菌或欧文氏菌来源，或培门冬酰胺酶）、CTX、阿糖胞苷（Ara-C）及甲氨蝶呤（MTX）等。常用的经典诱导方案主要有：ALL样方案[19]、BFM-90/95[20]、hyper-CVAD[21]、儿童样ALL方案[3]，常用的治疗方案见表4。另外对于身体虚弱或老年患者应结合患者的基本情况采用减低剂量的方案进行化疗。为降低复发率、提高生存率，诱导治疗结束后应尽快开始缓解后的巩固强化治疗，但最佳巩固治疗方案尚无统一意见，根据儿童ALL方案的设计，缓解后治疗可包括1～2个疗程再诱导方案（如VDLP方案），以MTX和Ara-C为基础的方案各2～4个疗程。推荐有条件的患者选择造血干细胞移植，自体造血干细胞移植（auto-HSCT）和异基因造血干细胞移植（allo-HSCT）各有利弊。除接受allo-HSCT的患者外，其余患者均应接受维持治疗，基本方案为：6-巯基嘌呤（6-MP）60～75 mg/m^2^每日1次，MTX 15～20 mg/m^2^每周1次。亦可选择POMP方案，每个月1次，共维持24个月。表观遗传学药物（如西达本胺）值得尝试，需要更多的临床研究来证明其有效性。

**表4:** 成人T淋巴母细胞淋巴瘤的常用化疗方案

方案	药物	剂量	用药时间
VDLP	泼尼松	60 mg/m^2^，口服	第1～28天减停
	长春新碱	1.5 mg/m^2^（最大剂量2 mg），静脉注射	第1、8、15、22天
	柔红霉素	30 mg/m^2^，静脉注射，持续1 h	第1、8、15、22天
	培门冬酶	2 500 IU/m^2^，肌肉注射，最大剂量3 750 IU	第2、16天
CAT	环磷酰胺	1 000 mg/m^2^，静脉注射	第1、15天
	阿糖胞苷	75 mg/m^2^，静脉注射	第3～6天、第17～20天
	6-巯基嘌呤	60 mg/m^2^，口服	第1～28天
Hyper-CVAD A方案	环磷酰胺	300 mg/m^2^，每12 h 1次，静脉注射（持续2 h以上）	第1～3天
	美司钠	600 mg·m^−2^·d^−1^，环磷酰胺用药前1 h至最后1次环磷酰胺后12 h	
	阿霉素	16.6 mg·m^−2^·d^−1^	第4～6天
	地塞米松	40 mg/d	第1～4天、第11～14天
	长春新碱	1.5 mg/m^2^（最大剂量2 mg）	第1、11天
Hyper-CVAD B方案	甲氨蝶呤	1 g/m^2^（亚叶酸钙解救）	第1天
	阿糖胞苷	3 g/m^2^，每12 h 1次，静脉注射（可根据患者年龄、体力情况、疾病状态酌情调整剂量）	第2～3天
POMP	6-巯基嘌呤	50 mg，口服	每日3次
	甲氨蝶呤	20 mg/m^2^，口服或静脉注射	每周1次
	长春新碱	2 mg，静脉注射	每月1次
	泼尼松	100 mg，口服	第1～5天

3. 放疗：纵隔是T-LBL最常见复发部位之一，纵隔放疗是降低纵隔复发的重要手段，但是由于纵隔放疗存在心脏损伤、甲状腺功能异常等不良反应，同时也可导致化疗的延迟，因此在成人的治疗方案中，纵隔放疗的必要性存在较大争议。有研究指出，在Hyper-CVAD方案的基础上加上纵隔放疗不仅可以降低患者纵隔复发的风险，亦可提高患者的生存率[22]；也有回顾性分析结果表明，纵隔放疗可显著降低患者纵隔复发的风险，但对患者的总生存（OS）率及DFS率无明显影响[23]。因此，基于降低患者纵隔复发的风险而言，纵隔放疗在成人T-LBL中仍值得推荐，而生存是否能够获益可能与纵隔放疗出现的严重并发症有关。对于原发结外或者淋巴结的患者，治疗结束后可考虑行受累区域放射治疗。

4. CNS预防：在NHL-BFM-90方案中，对所有初诊、CNS无侵犯的T-LBL患者均需行头颅12 Gy预防照射。但NHL-BFM-95方案中取消了CNS阴性患者的预防性头颅照射，且CNS复发亦未见增加，因此，目前T-LBL患者的治疗可采用鞘内注射化疗药物取代头颅预防照射。诱导治疗过程中没有CNS症状者可以在血细胞计数达安全水平后行鞘内注射。巩固强化治疗中也应进行积极的CNS预防，鞘内注射频率一般不超过2次/周（鞘注次数一般应达6次以上，高危组患者可达12次以上）。鞘内注射主要用药包括：地塞米松、MTX、Ara-C。常用剂量为MTX 10～15 mg/次、Ara-C 30～50 mg/次、地塞米松5～10 mg/次，三联（或两联）用药。

5. HSCT：auto-HSCT和allo-HSCT各有优劣，auto-HSCT复发率高，allo-HSCT治疗相关死亡率高，3～5年生存获益（DFS、OS）并无差异，因此，对于T-LBL患者优先选择何种移植方式仍不明确。推荐：①非第一次完全缓解（CR）状态、疾病更晚期或骨髓受累患者，应在充分评估安全性的基础上选择allo-HSCT，理由是allo-HSCT具有移植物抗肿瘤效应优势。②第一次CR且没有骨髓受累患者，优先推荐auto-HSCT。我国学者基于多中心临床样本构建了miRNA分子标签、DNA甲基化组学的综合模型，可精准鉴定出诱导化疗第一次CR后造血干细胞移植的获益人群[24]，辅助对移植方式的选择和评判。

auto-HSCT：T-LBL患者auto-HSCT后3年DFS率波动在62％左右，中高危患者的疗效显著低于低危患者，首次达到CR后接受auto-HSCT的患者疗效优于其他类型的患者[20],[25]–[28]；对中高危患者而言，复发是该类型患者移植失败的主要原因，改变移植预处理方案或移植策略（如双次auto-HSCT）可克服复发率高的临床问题，双次auto-HSCT的1年及3年复发率为12.1％和26.5％，3年无进展生存率为73.5％，OS率为76.3％，显著优于化疗及单次auto-HSCT，是值得推荐的疗法[29]–[30]。常用的预处理方案包括BEAM方案［卡莫司汀、依托泊苷（VP16）、Ara-C和美法仑］、BEAC方案（卡莫司汀、VP16、Ara-C和CTX）、CBV方案（CTX、VP16和卡莫司汀）和包含全身放射治疗（TBI）的方案，具体可参考《造血干细胞移植治疗淋巴瘤中国专家共识（2018版）》。

allo-HSCT：可显著降低患者的复发率，但各中心数据显示生存率差异较大，3年DFS率波动在39％～79％，平均约64％，移植相关死亡率为25％～28％，是影响疗效的重要因素，因此选择合适的移植患者和供者尤为重要，建议根据患者的年龄、一般情况、供者条件和移植风险评估综合评判[31]–[35]。

6. 复发难治（R/R）T-LBL的治疗：治疗方案优先推荐参加临床研究，再次诱导达到CR后行allo-HSCT，而化疗敏感的患者亦可以选择auto-HSCT。推荐的再诱导方案有：①以中大剂量Ara-C为主的联合化疗方案；②Hyper-CVAD；③新药联合化疗方案：如奈拉滨联合方案治疗R/R T-ALL/T-LBL患者，CR率36％，PR率14％[36]；维奈克拉组合方案，CR率61％[37]，维奈克拉、小剂量的Navitoclax联合化疗，CR率66.7％[38]；西达本胺联合方案，ORR 71％，CR率47％，两个疗程CR率达65％[39]；临床试验：靶向CD7的CAR-T细胞治疗采用无需基因编辑的自然选择CD7 CAR-T细胞治疗R/R T-ALL/LBL，94.12％的骨髓受累患者28 d实现了微小残留病阴性的CR[40]，可考虑桥接造血干细胞移植巩固疗效[41]；自体CD7 CAR-T细胞治疗8例R/R T-LBL患者，3个月缓解率87.5％[42]。

四、疗效评判

T-LBL疗效评判目前主要参考Lugano 2014淋巴瘤治疗效果评价标准。

近年来液体活检技术在淋巴瘤的诊断和辅助治疗选择中展现出独特的作用[43]，有条件的中心可开展液体活检的方法进行疾病状态的辅助监测和疗效评估，但尚需更多的循证医学证据。

五、治疗流程

为进一步规范成人T-LBL的治疗，本共识建议各中心按如下流程进行治疗（图1）。

**图1:**
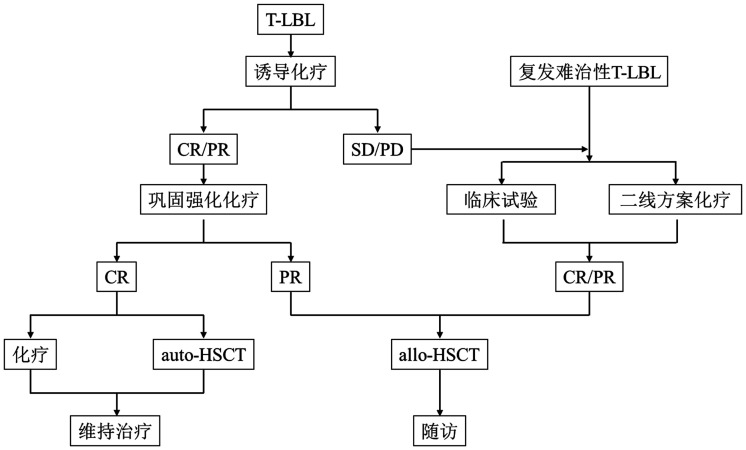
T淋巴母细胞淋巴瘤（T-LBL）治疗流程图 **注** CR：完全缓解；PR：部分缓解；SD：疾病稳定；PD：疾病进展
